# Editorial: Inborn errors of carbohydrate metabolism volume II

**DOI:** 10.3389/fgene.2026.1813006

**Published:** 2026-03-12

**Authors:** José Elías García-Ortíz, Ida Vanessa Doederlein-Schwartz, Melania Abreu-González, Iván Martínez-Duncker

**Affiliations:** 1 División de Genética, Centro de Investigación Biomédica de Occidente, Centro Médico Nacional de Occidente-Instituto Mexicano del Seguro Social (CMNO-IMSS), Guadalajara, Mexico; 2 Department of genetics, Universidade Federal do Rio Grande do Sul (UFRGS), Porto Alegre, Brazil; 3 Genos Médica Centro Especializado en Genética, Mexico City, México; 4 Centro Médico ABC, Mexico City, México; 5 Laboratorio de Glicobiología Humana y Diagnóstico Molecular, Centro de Investigación en Dinámica Celular, Instituto de Investigación en Ciencias Básicas y Aplicadas, Universidad Autónoma del Estado de Morelos, Cuernavaca, Mexico

**Keywords:** carbohydrate, CDG (congenital disorder of glycosylation), inborn error (disorder) of metabolism, lysosomal storage disease (LSD), metabolism, GLUT1, polyglucosan, LOPD

Inherited disorders of carbohydrate metabolism comprise a heterogeneous and expanding group of rare disorders that disrupt fundamental cellular processes, including glycosylation, glycogen metabolism, and glucose transport. Although individually rare, collectively these disorders represent a clinically significant and often underrecognized burden of neurological, neuromuscular, ophthalmologic, and systemic manifestations. Advances in genomic technologies, disease modeling, and population-level analyses are now reshaping how these conditions are diagnosed, understood, and managed.

This Research Topic represents volume II of *Inborn Errors of Carbohydrate Metabolism* and builds directly upon the conceptual and clinical framework established in volume I, which surveyed major domains across congenital disorders of glycosylation (CDG), lysosomal storage diseases, glycogen storage disorders, and disorders of glucose and galactose metabolism, spanning molecular mechanisms, diagnostic strategies, and emerging therapies.

The present volume focuses on phenotypic expansion, diagnostic pitfalls, tissue-specific disease mechanisms, and population-level impact, further illustrating the dynamic and evolving nature of this field. A recurring theme across the contributions is that genotype, biochemical defect, and clinical presentation do not always align in a predictable way, an issue that continues to challenge diagnosis, prognostication, and counseling. Several articles highlight that pathogenic variants in genes central to carbohydrate metabolism can present with a wide phenotypic spectrum, ranging from severe infantile encephalopathies to apparently asymptomatic adult presentations identified incidentally. These observations underscore the need for integrative diagnostic frameworks that extend beyond classical phenotype-based classifications.

Rare epileptic and neurodevelopmental phenotypes are prominently represented in this Research Topic. Alaamery et al. describe a founder variant in UDP-glucose dehydrogenase (UGDH), an enzyme that metabolizes UDP-glucose to UDP-glucuronic acid, a nucleotide sugar that is required for glycosaminoglycan/proteoglycan biosynthesis and broader UDP-glucuronic acid–dependent processes, critically involved in neural migration and connectivity during early brain development. The variant was associated with developmental epileptic encephalopathy in a large consanguineous Saudi family, expanding the role of glycosylation pathways in early brain development and neuronal connectivity. This work not only identifies a population-specific pathogenic variant but also highlights how defects in nucleotide sugar metabolism can converge on neurodevelopmental outcomes traditionally attributed to synaptic or ion channel dysfunction.

Complementing this, Wang et al. Report two neonatal cases of glucose transporter 1 deficiency syndrome (GLUT1DS), a rare inherited disorder classically characterized by three clinical symptoms: epilepsy, paroxysmal movement disorders, and developmental delays in motor and cognitive function. GLUT1DS is caused by pathogenic variants in *SLC2A1*, which codes for Glut1, the major glucose transporter in the blood-brain barrier, causing reduced glucose transport into the central nervous system. The reported individuals were initially misdiagnosed with bacterial meningitis, illustrating how metabolic diseases may masquerade as infectious or inflammatory disorders in early life. In this regard, persistent cerebrospinal fluid hypoglycorrhachia in infants with seizures, developmental delay, or lethargy despite normal systemic glucose and sterile cultures, even in the presence of fever or transient inflammation, should be considered a critical diagnostic clue for GLUT1DS. These cases further exemplifies how children presenting with acute or subacute neurometabolic manifestations should undergo comprehensive genetic sequencing as part of the diagnostic workup for inborn errors of metabolism, particularly when infectious evaluations are inconclusive. Early implementation of next-generation sequencing can prevent diagnostic delay and misclassification of metabolic disorders as infectious processes, allowing timely and targeted therapeutic interventions.

This Research Topic also addresses lysosomal and glycogen storage disorders, conditions in which carbohydrate metabolism intersects with neurodegeneration and multisystem disease. Zhu et al. present a non-Ashkenazi Chinese family with adult polyglucosan body disease (APBD) caused by compound heterozygous variants in *GBE1*, which encodes the glycogen branching enzyme 1, involved in the formation of branched glycogen. APBD leads to decreased glycogen branching and the formation of slender, insoluble polyglucosan bodies, which primarily accumulate in the central and peripheral nervous systems. While APBD has historically been associated with specific founder mutations in Ashkenazi Jewish populations, this study provides compelling evidence that large structural variants in *GBE1* can underlie late-onset, progressive neurodegenerative phenotypes when present in combination with a hypomorphic allele. The combination of the *GBE1* exon 3–7 deletion and the p. R156C pathogenic variant likely contributes to the delayed onset and slow progression observed in this case, supporting the concept that specific allelic configurations modulate residual enzymatic activity and, consequently, disease expressivity. By integrating detailed clinical phenotyping, next-generation sequencing–based CNV detection, and *in silico* protein structural modeling, the authors move beyond variant reporting to establish biological plausibility for pathogenicity. Equally important is the clinical insight offered by this study: adults presenting with slowly progressive spastic paraplegia, sensory neuropathy, and autonomic dysfunction may harbor undiagnosed carbohydrate metabolism disorders, particularly when standard neuromuscular or neurodegenerative workups are unrevealing.

Sciacco et al. present the first detailed molecular and biochemical characterization of a genetically confirmed homozygous *GAA* c.-32-13T>G (IVS1) late-onset Pompe disease (LOPD) case in a clinically asymptomatic adult, discovered incidentally during carrier screening. Although this splicing variant is the most common pathogenic allele in Caucasian LOPD patients, the spectrum of clinical expression — especially in homozygotes — has been poorly defined. The authors provide a comprehensive genotype–phenotype correlation, including transcript and protein analyses across multiple tissues, and show that marked biochemical abnormalities coexist with the complete absence of overt neuromuscular symptoms in adulthood, thereby challenging traditional genotype-guided assumptions about disease onset and severity. The most interesting and field-advancing highlight of this work is the dissociation between profound biochemical deficiency and clinical phenotype, demonstrating that severe reduction of GAA mRNA transcripts, including exon 2, and markedly diminished acid α-glucosidase activity can be present without neuromuscular or cardiopulmonary symptoms in a 33-year-old individual. This prompts a re-evaluation of prognostic assumptions and follow-up strategies in late-onset Pompe disease.

Translational and experimental approaches are exemplified by Cubilla et al., who develop an *in vitro* photoreceptor cell model to investigate the molecular basis of ophthalmic manifestations in ALG2-CDG, a type of CDG caused by pathogenic variants in *ALG2* that codes for an alpha-1,3-mannosyltransferase that catalyzes the second and third mannosylation steps in the N-linked glycosylation pathway. By linking a defined glycosylation defect to tissue-specific cellular vulnerability, this work exemplifies how disease modeling can bridge the gap between molecular pathology and organ-specific clinical outcomes. The main contribution of this study is the development and validation of a disease-relevant photoreceptor cell model to investigate tissue-specific pathogenic mechanisms in CDG, using ALG2-CDG as a proof of concept. Such models will be essential for evaluating targeted therapies in disorders where conventional biomarkers poorly reflect disease progression.

Importantly, this Research Topic extends beyond individual case studies to address population-level disease burden. Yu et al. leverage GBD 2021 data across 204 countries and territories to track G6PD deficiency from 1990 to 2021, documenting a marked rise in absolute prevalence to 443.3 million cases in 2021 (+80.17% vs. 1990) and pronounced geographic heterogeneity, with the highest case counts in South Asia and the largest relative increase in Andean Latin America.

It is also relevant to highlight the role of preventive reproductive genetics in the management of inherited metabolic disorders. Zagaynova et al. report an assisted reproduction + PGT-M strategy for a couple at risk of infantile GM1 gangliosidosis after identifying parental carriage of two *GLB1* variants (c.699delG and c.809A>C). Their workflow combined direct mutation testing with STR-based haplotyping flanking *GLB1*, enabling discrimination of carrier, affected, and unaffected embryos; an embryo free of disease-causing alleles was selected for transfer and the pregnancy resulted in the birth of a healthy child. This case concretely illustrates how PGT-M can prevent recurrence of severe autosomal recessive lysosomal disease and reduce reliance on later prenatal decision points.

Collectively, the articles in this Research Topic highlight several key directions for the field. First, phenotypic variability and incomplete penetrance are emerging as defining features rather than exceptions in disorders of carbohydrate metabolism. Second, early and accurate diagnosis—often reliant on subtle biochemical clues or broad genomic testing—is critical, particularly when disease-modifying therapies are available. Third, integrative approaches that combine clinical observation, functional modeling, and population-level data are essential for fully capturing disease mechanisms and impact.

As therapeutic pipelines expand, the insights provided by these studies will be increasingly relevant for precision medicine. This Research Topic underscores that advancing the field will require not only identifying new disease-causing variants but also rethinking how we define disease onset, severity, and treatability across the lifespan.

